# Temporal resolution of birth rate analysis in zooplankton and its implications for identifying strong interactions in ecology

**DOI:** 10.1002/ece3.10341

**Published:** 2023-07-24

**Authors:** Leonard V. Polishchuk, Anna A. Kasparson

**Affiliations:** ^1^ Department of General Ecology and Hydrobiology, Biological Faculty Lomonosov Moscow State University Moscow Russia; ^2^ Kharkevich Institute for Information Transmission Problems Russian Academy of Sciences Moscow Russia

**Keywords:** a single strong interaction's lifetime, bottom‐up effects, contribution analysis, Edmondson–Paloheimo model for birth rate, Liebig's law of the minimum, population dynamics, strong interactions in ecology, temporal resolution, zooplankton

## Abstract

Expanding on Haeckel's classical definition, ecology can be defined as the study of strong and weak interactions between the organism and the environment, hence the need for identifying strong interactions as major drivers of population and community dynamics. The solution to this problem is facilitated by the fact that the frequency distribution of interaction strengths is highly skewed, resulting in few or, according to Liebig's law of the minimum, just one strong interaction. However, a single strong interaction often remains elusive. One of the reasons may be that, due to the ever‐present dynamics of ecological systems, a single strong interaction is likely to exist only on relatively short time intervals, so methods with sufficient temporal resolution are required. In this paper, we study the temporal resolution of contribution analysis of birth rate in zooplankton, a method to assess the relative strength of bottom‐up (food) versus top‐down (predation) effects. Birth rate is estimated by the Edmondson–Paloheimo model. Our test system is a population of the cladoceran *Bosmina longirostris* inhabiting a small northern lake with few planktivorous predators, and thus likely controlled by food. We find that the method's temporal resolution in detecting bottom‐up effects corresponds well to the species' generation time, and the latter seems comparable to the lifetime of a single strong interaction. This enables one to capture a single strong interaction “on the fly,” right during its time of existence. We suggest that this feature, the temporal resolution of about the lifetime of a single strong interaction, may be a generally desirable property for any method, not only the one studied here, intended to identify and assess strong interactions. Success in disentangling strong interactions in ecological communities, and thus solving one of the key issues in ecology, may critically depend on the temporal resolution of the methods used.

## Graphical abstract

The temporal resolution of one of the methods to assess the relative strength of bottom‐up (food) versus top‐down (predation) effects in zooplankton is found to be comparable to the species' generation time and, approximately, to the lifetime of a single strong interaction. We suggest that dynamically‐oriented methods with a temporal resolution of about the lifetime of a single strong interaction may be key in disentangling strong interactions in ecological communities.

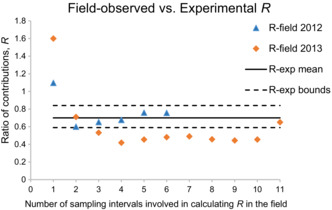

## 1 INTRODUCTION

Ecology has long been known as the science of interactions. This can be seen from the continuity of the two popular definitions separated by more than 100 years, namely that of Haeckel (1866: 286, quoted in Nikol'skii, 2014: 13–17) that ecology is the science of the relationships between the organism and its organic and inorganic environment and that of Krebs (1972, 2014) that ecology is the “scientific study of the interactions that determine the distribution and abundance of organisms”; common to both is the notion of interactions (or relationships). An important point to be added from more recent work is that ecological interactions are not of the same strength; some of them are much stronger than the others, resulting in a substantial nonzero variance in the interaction strength (Allesina & Tang, 2015; May, 1972; Pimm, 2002). The strong interactions are a major immediate driver of population and community dynamics (Paine, 1992). The weak interactions are no less important because, collectively, they play a primary role in the stability of ecological communities (de Ruiter et al., 1995; Downing et al., 2020; McCann et al., 1998). Expanding on Haeckel's definition, ecology now appears as the study of strong and weak interactions between the organism and the environment, and identifying strong interactions is one of its main goals.

A great help in solving this problem is that the frequency distribution of interaction strengths is not uniform; in fact, it is highly skewed such that there are few strong and many weak interactions, as first shown by Paine (1992) for a rocky intertidal community and corroborated by subsequent works (e.g., Jordán et al., 2003, Preston et al., 2018, 2019; reviewed in Landi et al., 2018, Power et al., 1996, Wootton & Emmerson, 2005). Remarkably (and perhaps surprisingly), the idea of “few strong – many weak interactions” may even be a quarter of a century older than Haeckel's (1866) definition of ecology. It can be considered to go back to Liebig's law of the minimum, according to which there is only one limiting factor, for example, a nutrient in shortest supply (Liebig, 1840, see also Nikol'skii, 2014; Berryman, 1993, 1999, 2003) provides a recent discussion of the law. The Liebig law can be viewed as the “few strong – many weak interactions” concept pushed to its limit: There is only one strong interaction, or equivalently a single limiting factor, and the rest are all weak. And vice versa, the concept can be regarded as a relaxed version of Liebig's law: As suggested by Berryman (1993), when the system is out of equilibrium, there may be not a single but a few strong interactions. It is this “few strong – many weak interactions” property that makes the task of identifying strong interactions feasible, for it is possible (or at least easier) to find one or a few strong effects but hardly so when there are many. Unfortunately, Liebig's law in both its strict (a single limiting factor) and relaxed (few strong–many weak interactions) forms acts as an existence theorem in mathematics: It posits the existence of a single or just a few strong interactions but says little about how to find them.

Contrary to expectations based on Liebig's law, a single strong effect often remains elusive. One of the reasons may be that temporal dynamics is a characteristic feature of ecological systems (e.g., Kerimoglu et al., 2013; reviewed in Leroux & Loreau, 2015), so individual strong interactions may operate over relatively short time intervals, and ability to identify them would depend in a major way on the temporal resolution of the methods used. To be more specific, in this study we focus on trophic interactions, namely bottom‐up (food availability) and top‐down (predation pressure) effects, in populations of zooplankton. These interactions play a pivotal role in the functioning of populations and communities in general (Leroux & Loreau, 2015) and in zooplankton in particular (Feniova et al., 2019; Gliwicz, 2003; Hampton et al., 2006; Huber & Gaedke, 2006; Liu et al., 2020; Marino et al., 2020). The key thing is that they tend to alternate over time. Thus, according to the seminal PEG (Plankton Ecology Group) model of seasonal succession in plankton communities (Sommer et al., 1986, 2012; Straile, 2015), in temperate waters a spring increase in herbivorous zooplankton is typically caused by an abundant food supply while a subsequent decline is affected by fish predation as well. These observations are in good agreement with the general theory of cascading top‐down effects (Leroux & Loreau, 2015; Oksanen et al., 1981) according to which food limitation and predation pressure would replace each other over time as major driving forces of population dynamics. Then, temporal averaging, which is a direct consequence of the insufficient temporal resolution of the methods, would result in bottom‐up and top‐down effects (or, for that matter, any factors) contributing roughly equally to population changes, giving a misleading and discouraging impression that “everything depends on everything else” (Berryman, 1993; in popular literature this formulation is known as Barry Commoner's first law). This problem is known by many names such as the “average temperature across the hospital” (Vilenkin, 1978) and, on a less ironic note, the fallacy of averaging (cf. Welsh et al., 1988), further emphasizing its importance. Our proposition is that the chance to detect a single governing factor or at least one clearly prevailing over the others would be higher if the method at hand has sufficient temporal resolution. Therefore, studying temporal resolution may be key to identifying strong interactions and thus to understanding population dynamics.

In this paper, we have studied the temporal resolution of one particular method, namely, contribution analysis of birth rate, which is intended to assess the relative strength of bottom‐up versus top‐down effects in cladoceran zooplankton (Polishchuk, 1995; Polishchuk et al., 2013). Birth rate is estimated by the Edmondson–Paloheimo model (Edmondson, 1968; Paloheimo, 1974). The model gives per capita birth rate as a function of fecundity (number of eggs per adult female), proportion of adults, and egg development rate. Fecundity and proportion of adults are taken as intermediaries between, respectively, food and predators, on the one hand, and birth rate, on the other. The effect of food and predators on birth rate and, by implication, on population dynamics is expressed as the product of the partial derivatives of birth rate with respect to fecundity and proportion of adults, respectively, times actual changes in fecundity and proportion of adults. The meaning of this calculation is that the partial derivatives yield a potential change in birth rate in response to a unit change in fecundity or proportion of adults, while the product of this potential change by the actual changes in fecundity and proportion of adults gives the component changes in birth rate caused by those population characteristics. After Caswell (1989, 2001: Ch. 10), who proposed this as a general approach, the products are termed contributions (of changes in fecundity and proportion of adults to the resulting change in birth rate), hence the name of the method, contribution analysis of birth rate (see Section 2 for further details).

The next step in developing contribution analysis of birth rate has been to construct the ratio of contributions of changes in the proportion of adults and fecundity to change in birth rate; we call it the ratio of contributions, *R* (Polishchuk et al., 2013). The contributions are taken in absolute value, so *R* ≥ 0. The behavior of *R* under different combinations of top‐down and bottom‐up effects has been studied experimentally (Polishchuk et al., 2013). The laboratory and computer experiments on *Daphnia* have shown that *R* < 1 is indicative of strong bottom‐up effects as compared to top‐down effects. These results were obtained by considering 6 or 11 sampling intervals in laboratory and computer experiments, respectively. No attempt was made to assess whether the instructive values of the ratio of contributions could be obtained on fewer intervals, a question that is directly related to the temporal resolution of the method. Thus, contribution analysis of birth rate has the potential to identify strong bottom‐up effects, but neither have the above experimental findings been tested in the field nor has the method's temporal resolution been examined.

In this study, we are dealing with a herbivorous zooplankton species, the cladoceran *Bosmina longirostris*, inhabiting a small northern lake with few planktivorous predators and thus likely controlled by food. We describe this situation as one in which *strong* bottom‐up effects are at place, based on the general theory of cascading top‐down effects (Leroux & Loreau, 2015; Oksanen et al., 1981) according to which bottom‐up and top‐down effects are inextricably linked and tend to negatively correlate with one another (see above). Since predation effects on our *Bosmina* population are weak, bottom‐up effects are to be strong (except for the initial phase of population growth, see Section 3). Note that in the previous experimental work (Polishchuk et al., 2013) to which we compare the results of the present study, strong bottom‐up effects were defined in the same way: They were considered strong in microcosms (aquaria) where there was no predation other than sampling.

Using this *Bosmina* population as a test case, we pursue two goals. First, we test whether contribution analysis of birth rate allows one to detect strong bottom‐up effects in natural zooplankton populations. More specifically, we test the prediction, based on the independent experimental findings (Polishchuk et al., 2013), that under strong bottom‐up effects the ratio of contributions *R* is less than 1. Second, and most important, we determine the temporal resolution of contribution analysis of birth rate; that is, the minimum number of sampling intervals and the minimum length of time needed to reliably determine *R* and thus to identify strong bottom‐up effects. Although the problem of temporal resolution is addressed here through one particular example, we believe it is a general one. Given that ecological systems typically vary over time and can move rather quickly from the state of being controlled by one factor to the state of being controlled by another, success in identifying strong interactions may very much depend on the temporal resolution of the method used.

## 2 MATERIALS AND METHODS

### 2.1 Study site and ecological environment

Data were collected at Vodoprovodnoye Lake, which is located near the Arctic Circle, in the vicinity of the White Sea Biological Station of Lomonosov Moscow State University. It is a small shallow freshwater lake with a maximum depth of about 2.7 m and a surface area of about 0.6 ha (Bizina, 2000; Mardashova et al., 2020; A. N. Pantyulin, personal communication). Secchi disk transparency regularly recorded during the study period in 2013 was 1.4 ± 0.2 m (mean ± SD; *n* = 23), which suggests a eutrophic state (Bigham Stephens et al., 2015). The lake is surrounded by forest and in some places by floating mats of peat mosses, and the water has a yellowish tint, likely due to the presence of humic substances. It is known that in colored shallow lakes such as this one Secchi disk depth may not be indicative of trophic state (Carlson, 2007).

**FIGURE 1:**
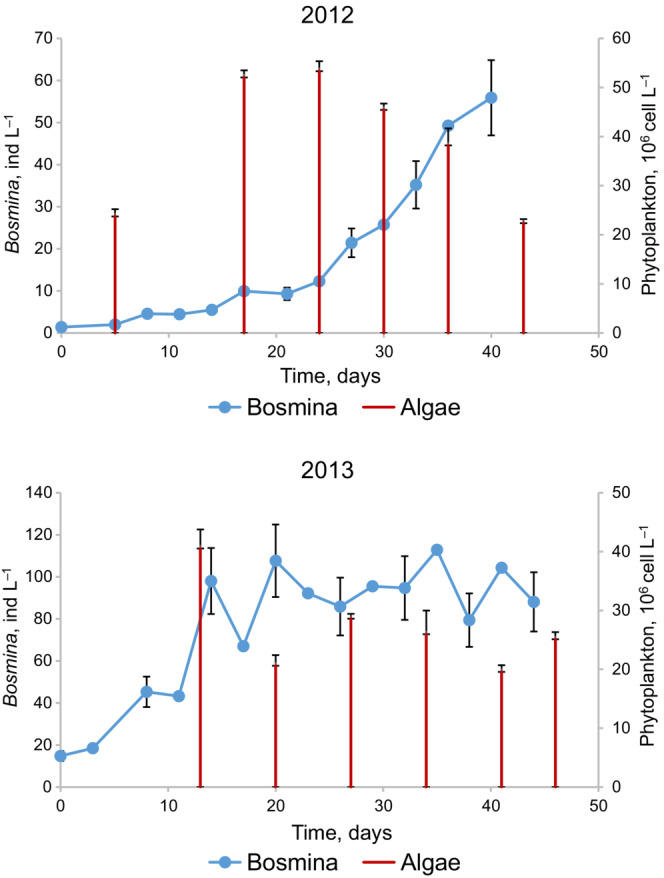
Changes in *Bosmina* population density and phytoplankton abundance (size <20 μm) in 2012 and 2013, and statistical errors. For *Bosmina* density, the error is associated with horizontal heterogeneity and based on three in 2012 and five in 2013 samples taken from different sites once per season. The relative standard error (SE/mean) was the same in both years and equal to 0.16; errors are calculated as 0.16 times population density on a given date. To avoid cluttering, error bars are shown for samples with odd numbers only. At the beginning of the season in 2012, error bars are not seen because they are smaller than the symbol size. For phytoplankton, the error is associated with sample counting; only positive error bars (+1 SE) are shown.

On two occasions in 2013, we measured nutrient concentrations in the lake (Table 1). According to the classification by Oksiyuk and Zhukinsky (Oksiyuk et al., 1993), which is popular in Russia and neighboring countries, the levels of phosphate and nitrate suggest that the lake is oligotrophic, while the levels of nitrite and ammonium correspond to a mesotrophic or a eutrophic state, respectively. Based on the lowest trophic status (because it is determined by a nutrient that acts as a limiting factor—and this is again a reminder of Liebig's law), the lake is likely to be oligotrophic. This conclusion is consistent with the Russian guidelines for aquatic ecosystems (*On the standards for phosphate (MPC) in fishery water bodies* 2015 URL: http://www.vniro.ru/ru/novosti/o‐rybokhozyajstvennom‐normative‐pdk‐na‐fosfaty, Accessed on 20 July 2021) that stipulate that phosphate concentrations of less than 0.05 mg P L^−1^ indicate an oligotrophic state of the water body. Although in our case both phosphate and nitrate implicate an oligotrophic state, the latter has a readily available source of replenishment (ammonium) while the former does not; hence, the lake is likely to be P‐limited. Finally, concentrations of algal pico‐ and nanoplankton observed in Vodoprovodnoye Lake during both study seasons, 2012 and 2013 (see Figure 1), were comparable to those reported for oligotrophic lakes (Stockner & Shortreed, 1989).

**TABLE 1:** Nutrient concentrations in Vodoprovodnoye Lake.

Date	Depth, m	PO_4_^3−^, mg P L^−1^	NH_4_^+^, mg N L^−1^	NO_2_^−^, mg N L^−1^	NO_3_^−^, mg N L^−1^
2 Jul 2013	1.0	0.004^a^	0.53 ± 0.09	0.006 ± 0.001	0.09 ± 0.01
21 Aug 2013	0.5	0.008^a^	1.32 ± 0.14	0.006 ± 0.001	0.12 ± 0.01

*Note*: The concentrations (±SE) are presented as P (for phosphate) and N (for ammonium, nitrite, and nitrate), which are calculated from the raw concentrations of the ions based on the stoichiometry. The measurements were performed by the water quality testing laboratory, Kandalakshavodokanal, Kandalaksha.

^a^ Nominal value, as measured; below the detection threshold of 0.016 mg P L^−1^ of the method used.

An interesting property of the vertical temperature distribution in this shallow lake (though probably not uncommon for small forest Karelian lakes, Stepanova et al., 2010, but not found in some others, for example, in a nearby Verkhneye Lake) is that below 1 m temperature dropped sharply. Based on 23 series of vertical temperature distributions measured by us to a depth of 2 m in 2013 and a few more measurements at deeper depths made by A. N. Pantyulin (personal communication) with a YSI CastAway‐CTD probe (YSI Inc./Xylem Inc., USA), an average temperature profile was such that at a temperature of 19.7°C just below the surface, temperature at depths of 0.5 and 1 m was 18.6 and 16.5°C, respectively, while at depths of 1.5, 2, and 2.6–2.7 m it was 12.9, 10.0, and between 7 and 10°C, respectively. Ten vertical distribution surveys of *Bosmina* conducted from 25 June to 6 August 2013 showed that, on average, 89% of its animals, and approximately the same proportion of eggs, inhabited the upper 1‐m layer; hence, a large temperature gradient below this depth hardly had any influence on the population characteristics such as egg development time needed to calculate birth rate.

In 2012 and 2013 when we conducted our research, the only mesozooplankton species found in the pelagial of the lake were the cladoceran *Bosmina longirostris*, which is the focus of this study, and the rotifer *Asplanchna priodonta* (cf. Bizina, 2000). Given that *Asplanchna* is a predator (on small rotifers) and occasionally a grazer but in the latter case it feeds on large algae that cannot be ingested by *Bosmina* (Kappes et al., 2000), *Bosmina* had no species to compete with and thus may have experienced only intraspecific competition.

Among invertebrate predators that could potentially affect the cladoceran abundance, only *Chaoborus* larvae were present in the lake. However, there were so few of them, just about a dozen caught in the net during the entire two‐season study period, that they seem unlikely to have had any significant effect on the *Bosmina* population. Note that *Chaoborus* were found in the gut of the ninespine stickleback (see below), but this does not necessarily show that they were abundant in the lake; rather, this may indicate that the fish fed selectively on them.

The only fish predator present in the lake was the ninespine stickleback (*Pungitius pungitius*). The stickleback invaded Vodoprovodnoye Lake after 1995, when it was temporarily connected to a nearby Verkhneye Lake by a ditch (Bizina, 2000). To estimate its pressure on the *Bosmina* population, in 2013 we used minnow traps baited with “*TetraCichlid*” fish food (Tetra, USA). The traps were placed along the shore on two occasions, three traps from 29 June to 3 July and 10 traps on 17 July for 24 h, the latter case corresponding to high *Bosmina* abundance (see Figure 1 where 17 July is Day 24). Overall, just four stickleback individuals were caught—two on 3 July and two on 17 July, with body length and wet weight (mean ± SE) of 42.2 ± 1.3 mm and 0.90 ± 0.15 g. The analysis of fish gut contents showed that the ration predominantly consisted of insect remains, including *Chaoborus* larvae, and cladocerans, mainly Chydoridae. Only one specimen among several tens of items found resembled *Bosmina*. Based on this, we concluded that the *Bosmina* population did not experience any substantial predation pressure in Vodoprovodnoye Lake. Moreover, previously published data on zooplankton community dynamics in this lake (Bizina, 2000) suggest that even in 1997, when larger cladocerans were significantly affected by the then‐recent invasion by the stickleback, *Bosmina* largely escaped its impact.

### 2.2 Data collection

Zooplankton samples were collected every 3 days (very rarely 4 or 5 days) during both study periods: from 25 June to 4 August in 2012 and from 23 June to 6 August in 2013. The first date of sampling is further referred to as Day 0. Samples were collected from five stations distributed approximately evenly over the area of our small lake, using a plankton net (mesh size of 140 μm, mouth diameter of 14 cm) towed vertically from near the bottom to the surface. The five samples, one per station, were mixed together and then concentrated to the volume of 500 mL. This mixed sample was transferred to the laboratory for further analysis. The statistical error of *Bosmina* population‐density estimates caused by horizontal heterogeneity was assessed from two surveys conducted on 7 August 2012 and on 28 June 2013 and consisted of three and five samples, respectively, with each sample treated individually.

On every sampling occasion, we measured water temperature at three depths of 0.1, 0.5, and 1 m from the water surface. Given that below 1 m *Bosmina* were very few (see above), to assess their egg development time, which is temperature‐dependent (Bottrell et al., 1976), we used the average temperature at the abovementioned depths.

To count animals and eggs, a 5‐ to 20‐mL subsample of the total sample, depending on the abundance of zooplankton, was placed in a Bogorov tray, fixed with several drops of 4% buffered formalin and counted at 32× with a stereomicroscope. Only female *Bosmina* were found in the samples. Juvenile and adult bosminids were counted separately. The distinction was made on the basis of body size (determined with an ocular micrometer to the nearest 50 μm) and the presence of brood chamber, though detailed measurements were performed only in 2013. Usually, no less than 300 individuals were counted on every sampling occasion, except for the beginning of the 2012 study period, when *Bosmina* abundance was very low. The number of eggs in the brood chamber of adults was also determined. We thus obtained the number (expressed per liter) of eggs *E*, juveniles *N*_j_, and adults *N*_a_ on every sampling date. Finally, based on the water temperature and using a general formula from Bottrell et al. (1976: 445), equation for cladocerans other than daphnids, we calculated egg development time *D* (in days) and its reciprocal, the egg development rate *V* = 1/*D* ([*V*] = day^−1^). These parameters, *E*, *N*_j_, *N*_a_, *V* = 1/*D* plus the total population density *N* = *N*_j_ + *N*_a_, are all that we need to calculate birth rate.

Phytoplankton samples were collected approximately once a week using a 1‐L Ruttner sampler at depths of 0.1, 0.5, and 1 m below the water surface. Three phytoplankton samples, one from each layer at a single sampling site, were mixed and 1 L of the resulting sample was fixed with Lugol's iodine and decanted 2 weeks after the fixation. The samples were then analyzed using a Nageotte counting chamber under a Zeiss Axiovert 200 microscope (Carl Zeiss). The samples were dominated by cyanobacteria and small unidentified cells of cocci and flagellates with a cell size of less than 10 μm in diameter. Some larger flagellates (*Peridinium* spp.) were also found, though in low numbers. Only pico‐ and nanoplankton fractions (size <20 μm) were considered edible for *Bosmina*. The standard error of the edible plankton concentration was estimated on the basis of counts of three subsamples taken from the sample pertinent to a given date.

### 2.3 Calculating birth rate

The original Edmondson–Paloheimo model (Edmondson, 1968, his equation 13; Paloheimo, 1974) to calculate per capita birth rate *b* in zooplankton, in particular in cladocerans, is

$$b=\left(1/D\right)\;\ln\left(1+E/N\right)$$

To make the model better suitable for contribution analysis, it can be equivalently rewritten in terms of egg development rate *V*, fecundity *F* = *E*/*N*_a_ and proportion of adults *A* = *N*_a_/*N* (Polishchuk, 1995).

(1) $$b=V\;\ln\left(1+\mathrm{FA}\right)$$

The Edmondson–Paloheimo model is based on rather general assumptions (Polishchuk et al., 2013; Voronov, 1991) and thus describes birth rate quite well (Mooij et al., 2003, and references therein). Written in form (1), it allows one to relate birth rate to the quantity and quality of food (through *F*), predation pressure (through *A*; because in zooplankton predators are typically size‐selective), and temperature (through *V*; Polishchuk, 1995; Polishchuk et al., 2013). Thus, the Edmondson–Paloheimo model written in form (1) helps to build the bridge from the major environmental factors (food, or bottom‐up effect; predation, or top‐down effect; and temperature) through the mediation of the population characteristics (*F*, *A*, *V*, respectively) to birth rate.

### 2.4 Calculating contributions

Contribution analysis (Caswell, 1989) as applied to the Edmondson–Paloheimo birth rate model is a method to quantify the effect of changes in *F*, *A*, and *V* on the resulting change in *b* and thus to express the effects of the environmental factors that underlie *F*, *A*, and *V* on population dynamics, using birth rate (or to be more precise, change in birth rate) as a common currency (Polishchuk, 1995, Polishchuk et al., 2013). The basic equation of contribution analysis of birth rate is

(2) $$\Delta b=\frac{\mathrm{VA}}{1+\mathrm{FA}}\Delta F+\frac{\mathrm{VF}}{1+\mathrm{FA}}\Delta A+\left[\ln\left(1+\mathrm{FA}\right)\right]\Delta V$$

where the first term on the right‐hand side is the contribution of a change in fecundity Δ*F* to the resulting change in birth rate Δ*b* and similarly for the second and third terms associated with the contributions of Δ*A* and Δ*V*. The sum on the right‐hand side, however, equals Δ*b* only if shifts in *F*, *A*, and *V* are all sufficiently small. Often this will not be the case because those shifts represent actual population changes between successive sampling dates. To overcome this problem, we rewrite Equation (2) in terms of the derivatives with respect to time *t*

(3) $$\frac{db}{dt}=\frac{\mathrm{VA}}{1+\mathrm{FA}}\cdot \frac{dF}{dt}+\frac{\mathrm{VF}}{1+\mathrm{FA}}\cdot \frac{dA}{dt}+\ln\left(1+\mathrm{FA}\right)\cdot \frac{dV}{dt}$$

and integrate the left‐hand side and, term by term, the right‐hand side of Equation (3) over sampling interval *T*. To make the result tractable, we assume that *F*, *A*, and *V* vary linearly with time within *T* and arrive at the following expressions for contributions (Polishchuk et al., 2013)

(4) $$\mathrm{Con}F=\left[\frac{1}{T}\int_{0}^{T}\frac{\mathrm{VA}}{1+\mathrm{FA}}dt\right]\cdot \Delta F$$

(5) $$\mathrm{Con}A=\left[\frac{1}{T}\int_{0}^{T}\frac{\mathrm{VF}}{1+\mathrm{FA}}dt\right]\cdot \Delta A$$

(6) $$\mathrm{Con}V=\left[\frac{1}{T}\int_{0}^{T}\ln\left(1+\mathrm{FA}\right)dt\right]\cdot \Delta V$$

These integral contributions (for which we keep the same notation as for their nonintegral counterparts) involve *actual* changes in the corresponding population characteristics, Δ*F*, Δ*A*, Δ*V*, over the sampling interval *T*, and sum up exactly to the *actual* change in birth rate in that interval:

Δb=ConF+ConA+ConV

Also, the so‐defined contributions keep Caswell's (1989) original meaning as the product of the partial derivative times the actual change in the variable of interest. The only difference between the original definition and the contributions from Equations ((3), (4), (5)) is that the instantaneous partial derivative is replaced with its average value over *T* (the expressions in square brackets). This does not change the meaning of contributions.

It may be of interest to note that the above approach is a general one: it does not depend on the form of a particular function to be decomposed into contributions. It is always possible to replace instantaneous partial derivatives with the average values, thus making the sum of contributions equal to the resulting change in the function of interest.

Quantitatively, contributions from Equations ((4), (5), (6)) were obtained through numerical integration using a computer code written in QBasic (Appendix S1; the credit for the invention of the integral form of the contributions belongs to D. A. Voronov, who also wrote the QBasic code; for more details about the integration see Polishchuk et al., 2013).

### 2.5 Calculating the ratio of contributions

In order to detect the presence, and perhaps to measure the strength, of bottom‐up effects in the lake, we employ the ratio of contributions, one of which, that from Equation (5), is associated with change in the proportion of adults and the other, that from Equation (4), with change in fecundity. Those contributions refer to a certain sampling interval. Because a single interval may not be sufficient to obtain reliable estimates of the ratio, it is calculated over a time period spanning multiple consecutive sampling intervals. We have used the following 4‐step procedure to find the characteristic ratio of contributions over multiple sampling intervals (Polishchuk et al., 2013): (1) Contributions per sampling interval from Equations (4) and (5) are divided by the length of the interval (in days) to be expressed per day, denoted as (Con*F*)_day_ and (Con*A*)_day_. (2) The per day contributions are taken by absolute value (they are negative when ∆*F* or ∆*A* < 0), denoted as |(Con*F*)_day_| and |(Con*A*)_day_|. (3) For a set of sampling intervals involved in the time period of interest and the corresponding set of contributions, the median of |(Con*F*)_day_| and the median of |(Con*A*)_day_| are found. (4) The median |(Con*A*)_day_| is divided by the median |(Con*F*)_day_| to yield the ratio of contributions *R* for the time period of interest.

In this study, our ultimate goal is to determine the temporal resolution of the method, that is, the minimum number of sampling intervals sufficient to obtain a reliable and informative, in the context of detecting bottom‐up effects, ratio of contributions (of course, we did not know in advance if this number would exist). We thus calculate *R* for all individual sampling intervals, for all consecutive intervals taken in twos, including the overlapping pairs (i.e., (interval 1 and interval 2), (interval 2 and interval 3), and so on), for all consecutive intervals taken in threes, including the overlapping triplets (i.e., (interval 1, interval 2, and interval 3), (interval 2, interval 3, and interval 4), and so on), and so on up to a single complete set of intervals suitable for analysis in a given year (these are 6 and 11 last sampling intervals in 2012 and 2013, respectively; see Section 3). We then take the average of each set of *R* pertinent to a certain number of sampling intervals to get the *R*‐value to be associated with that number. Because many of the pairs, triplets, etc., of intervals overlap with each other, the individual *R*‐values cannot be considered independent in the statistical sense. Hence, we cannot and do not evaluate the statistical significance of the difference in *R* associated with different number of intervals. Instead, we compare our field estimates of *R* with the laboratory values obtained in the independent experiments (Polishchuk et al., 2013).

## 3 RESULTS

Although the *Bosmina* population did not experience any substantial predation pressure and hence top‐down effects were weak (see Section 2.1), it does not necessarily follow that bottom‐up effects were invariably strong. In the first part of the season when population sizes were relatively low, food may have been in excess, with no considerable bottom‐up effects to occur. Hence, our first task was, focusing on the second part of the season, to identify the periods of food shortage when bottom‐up effects were likely to be at work. These were the periods for which we calculated the ratio of contributions and compared it with the previously obtained experimental results.

**FIGURE 2:**
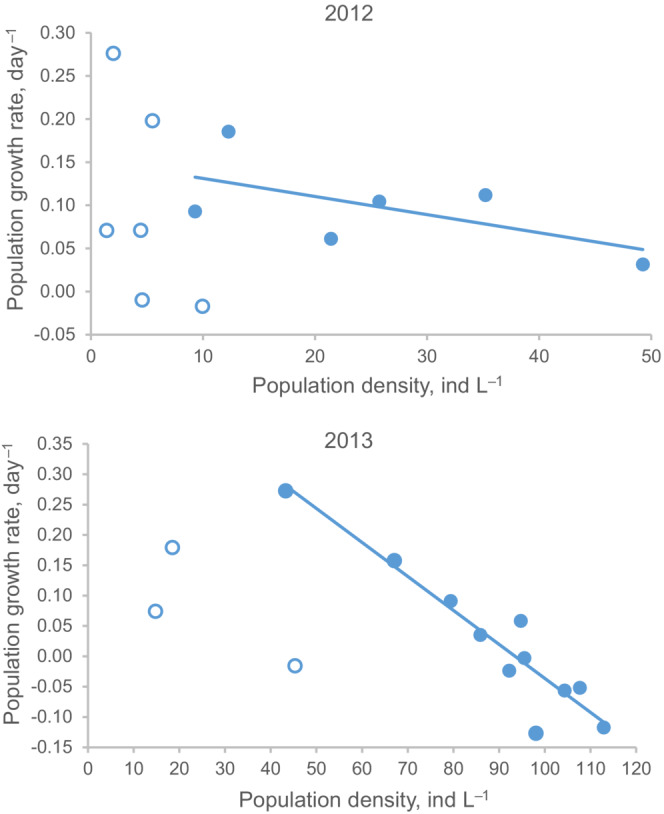
Relationships between *Bosmina* per capita population growth rate *r* and population density *N* in 2012 and 2013. Regression lines are based on the last 6 and 11 sampling intervals in 2012 and 2013, respectively. Data points included in the regressions are indicated by filled circles, those not included by empty circles. Regression equations are: *r* = −.0021 *N* + 0.15 (*p* = .20, *ρ* ^2^ = 0.37) in 2012 and *r* = −.0056 *N* + 0.52 (*p* < 10^−4^, *ρ* ^2^ = 0.88) in 2013.

**FIGURE 3:**
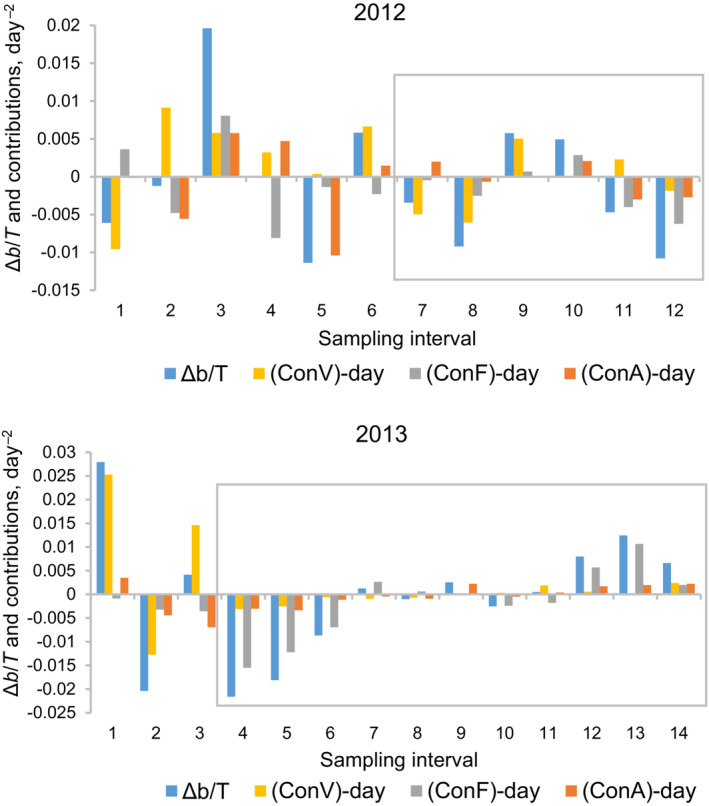
Birth rate analysis in *Bosmina longirostris* in 2012 and 2013. The analysis consists of decomposing changes in per capita birth rate *b*, Δ*b*, into three contributions, each due to changes in only one population characteristic, namely egg development rate, Con*V*, fecundity, Con*F*, or proportion of adults, Con*A*, and then calculating the ratio of contributions, |Con*A*| over |Con*F*| (see Figure 4). Each cluster of bars represents the decomposition of Δ*b* for a given sampling interval. In every cluster, the left‐most bar indicates Δ*b* divided by the length of the interval, *T*, in days; and the other three bars are contributions expressed on a per day basis. The contributions add up to the change in birth rate, that is, (Con*V*)_day_ + (Con*F*)_day_ + (Con*A*)_day_ = Δ*b*/*T*. Note that bars above or below the zero line reflect an increase or decrease in the corresponding characteristic in a given sampling interval. Rectangles delineate the periods in the second part of the season where density dependence manifests itself most clearly, and thus, bottom‐up effects are likely to be most pronounced. The ratio of contributions shown in Figure 4 refers to just these periods.

Given little predation pressure, periods of food shortage are those where negative density dependence manifests itself (Hixon & Johnson, 2009). The negative density dependence is defined as a negative relationship between per capita population growth rate *r* = [ln*N*(*t* + *T*)‐ln*N*(*t*)]/*T* and population size (density) *N*(*t*). Our *Bosmina* population was steadily increasing with some slowing down at the end in 2012 and showed a clear leveling‐off trend in 2013 (Figure 1). This difference may be due to lower *Bosmina* population densities in 2012 (*N* < 60 ind L^−1^ throughout the season) than in 2013 (*N* > 80 ind L^−1^ on the plateau), complemented with higher phytoplankton densities in the former year than in the latter (Figure 1). Visual inspection shows that relying on the last six sampling intervals (out of 12) in 2012 and the last 11 sampling intervals (out of 14) in 2013 gives a reasonably good linear *r*‐*N* relationship (Figure 2). Not surprisingly in light of the difference between the years discussed above, this relationship was weaker in 2012 (slope = −0.0021 ± 0.0014, *ρ* ^2^ = 0.37, *p* = .20) than in 2013 (slope = −0.0056 ± 0.0007, *ρ* ^2^ = 0.88, *p* < 10^−4^). Though in 2013 the relationship is nominally significant, a more rigorous analysis indicates that there is a 15% probability that it may result from measurement error. (This estimate is based on the following (Doncaster, 2006; Sibly et al., 2005): (1) due to measurement error the regression of *r* on ln*N*(*t*) must be linear and have a slope of −1/*T*; (2) in 2013, given that sampling interval is on average 3.1 days, −1/*T* = −0.32 day^−1^; (3) in 2013, the slope of the regression of *r* on ln*N*(*t*) is −0.407 ± 0.055 day^−1^; and (4) this yields approximately an 85% probability that the −0.32 lies beyond the range of the slope and a 15% that it lies within that range.) Thus, although our analysis does not permit us to establish with complete certainty the density dependence, it reveals the periods where this dependence and thus bottom‐up effects are likely to have been more pronounced, and in addition, suggests that these effects were stronger in 2013 than in 2012. The ratio of contributions was calculated just for these periods (Figure 3; the periods of interest are delineated with rectangles). It must be emphasized that once the periods had been defined, they were never tinkered around to revise the results of contribution analysis.

**FIGURE 4:**
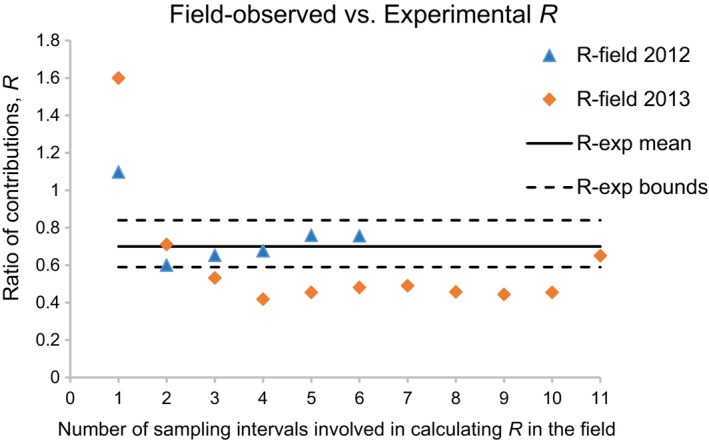
Comparison of the ratio of contributions *R* observed in a field population of *Bosmina longirostris* in 2012 (R‐field 2012) and 2013 (R‐field 2013) in this study, and in the experimental laboratory populations of *Daphnia galeata* in a separate work (R‐exp mean, the mean experimental value; Polishchuk et al., 2013). Common to the field and laboratory populations is the key role of food in population dynamics; hence, the general agreement between field and laboratory *R*‐values, seen in the graph starting from two sampling intervals, is evidence that *R* is an indicator of trophic conditions in the field. The graph also shows that two to four sampling intervals ensure minimum averaging needed to obtain the ratio of contributions characteristic of a given year. This indicates the temporal resolution of the method. The ratio of contributions in the field refers to 6 and 11 last sampling intervals in 2012 and 2013, respectively, a period where bottom‐up effects are likely to be most pronounced. The ratio of contributions observed in the laboratory is provided with 1 SE error bounds, R‐exp bounds; note that the upper and lower bounds are not exactly symmetrical about the mean because they are back‐calculated from the log‐transformed *R*.

Our second task was to calculate the average ratio of contributions in relation to the number of sampling intervals it is based on (we call it an *R* curve) and to compare this field value with the value from the laboratory experiments in which bottom‐up effects were implemented (Polishchuk et al., 2013). For ease of terminology, a set of consecutive sampling intervals is called below the time interval, and the time interval length is measured in terms of the number of sampling intervals involved. The pattern apparently common to both years is that the average ratio of contributions is greater than 1 at the time interval of length 1 (i.e., when sampling intervals are taken singly), drops to 0.6–0.7 at the time interval of length 2 (sampling intervals are taken in twos), and then generally levels off starting from either interval of length 2 in 2012 or intervals of length 3 or 4 in 2013, though in 2012 the plateau is less pronounced (Figure 4). A more objective criterion to locate the plateau can be derived by considering, in relation to our field *R*‐values, a ±1 SE band around the mean experimental value of *R* known from the independent experiments (Polishchuk et al., 2013). In 2012, starting from the interval of length 2, all field *R*‐values are found within the experimental error bounds (Figure 4). The fact that variability in *R* is limited by predefined boundaries proves the existence of an *R* plateau. In 2013, the bulk of *R*‐values, namely, those from the intervals of length 3 or 4 to the interval of length 10, comprise an almost perfect horizontal line, which goes parallel to the error band and thus forms a plateau (Figure 4).

The major difference between the years is the level of the plateau. In 2012, the ratio of contributions averaged over the plateau is *R* = 0.69 ± 0.03 (mean ± SE over time intervals of length 2–6), which is in excellent agreement with the experimental results (*R*_exp_ = 0.70; Polishchuk et al., 2013). In 2013, the ratio of contributions converges to a lower value of *R* = 0.47 ± 0.01 or *R* = 0.46 ± 0.01 (mean ± SE over time intervals of length 3–10 or 4–10, respectively). Moreover, in that year from interval of length 3 on, all *R*‐values lie below the lower experimental error bound (except for the value pertinent to 11 sampling intervals, which is excluded from the calculation of the averages above, see Figure 4). Thus, in both years *R* curves follow a declining plateauing pattern, and the plateau is lower in 2013 than in 2012.

That the *R* plateau in 2012 coincides with the experimental results shows that, first, it is the plateau *R*‐values that are indicative of bottom‐up effects, and second, the ratio of contributions of about *R* = 0.7 indicates bottom‐up effects to be at work. Next, to explain the difference between the plateau *R‐*values in 2012 and in 2013, we notice that low ratios of contributions, rather than high ones, point toward a food shortage (while higher ratios point to fish pressure; Polishchuk et al., 2013). Thus, the lower *R*‐values observed in 2013 suggest stronger bottom‐up effects in that year, which is in line with our finding of stronger density dependence in 2013 than in 2012. We conclude that the field *R*‐values are consistent with the experimental results not only in 2012 where they agree quantitatively but also in 2013 where they agree qualitatively.

Our third task was to determine the temporal resolution of the method. The temporal resolution is defined as the minimum number of sampling intervals, and the length of the corresponding time interval, which is sufficient to detect the presence, and perhaps to assess the strength, of bottom‐up effects. This minimum number is the one where the plateau in the *R* curve starts. In 2012, two sampling intervals would suffice to calculate the ratio of contributions that reveals bottom‐up effects, whereas in 2013 three or four sampling intervals are required to obtain *R‐*values indicative of stronger bottom‐up effects in that year than in 2012 (Figure 4). Given that the average sampling interval is 3.3 and 3.1 days in 2012 and 2013, respectively, the temporal resolution of the method is two to four sampling intervals or approximately 6–12 days.

## 4 DISCUSSION

In this study, we tested in the field a method of analysis of birth rate dynamics in zooplankton, termed contribution analysis of birth rate, and its derived metric, the ratio of contributions, *R*. To be more specific, we tested in the field whether *R* is indicative of strong bottom‐up effects when top‐down effects are weak or virtually absent. Furthermore—and this is perhaps even more important—we determined the temporal resolution of the method. The previous laboratory and computer experiments (Polishchuk et al., 2013) suggested that the *R* ratio might be useful to assess the relative strength of top‐down versus bottom‐up effects. The lake we worked on is a simple ecosystem with few planktivorous predators; that is, only bottom‐up effects are likely to be at work there. The system is further simplified by there being only one common species of crustacean zooplankton in the pelagial, the cladoceran *Bosmina longirostris*, which is the subject of this study. A less complex environment seems more conducive to testing the method, since it tends to be less “noisy,” thus making the cause‐and‐effect relationships, here the presumed effect of food on *R*, more straightforward; hence our choice of the lake. In the experiments, strong food effects were created, albeit with a different cladoceran species (*Daphnia galeata*), and the ratio of contributions *R* was determined. We use this experimental ratio as a point of reference with which to compare our field results.

It should be noted that in the experiments temperature was maintained constant while in the lake it was of course not. The question then arises whether the effect of temperature can be a source of discrepancy between the experimental and the field results. In short, the answer seems to be no. The most obvious reason is that, over the periods for which the contributions were computed (see Section 3 and Figure 3), in 1 year the average temperature happened to be nearly the same as in the experiments, though in the other year the difference was not that small (temperature in the experiments: 17.5°C; temperature in the lake (mean ± SE): 15.4 ± 0.3°C, *n* = 7, in 2012 and 17.6 ± 0.4°C, *n* = 12, in 2013). The second reason is a more fundamental one. Back in 1978, Winfried Lampert has suggested that fecundity may not be affected by temperature. Here, we clarify and expand his argument. A change in temperature is known to scale uniformly the duration of various developmental stages (Gillooly et al., 2002). When the temperature declines and a given stage lengthens, it accumulates more individuals in proportion to the increase in its length; that is, in the stage of eggs, juveniles and adults there occurs an increase in the number of eggs, juveniles, and adults, all by the same factor. (Similarly, when the temperature rises and the stages shorten, the respective abundances will uniformly shrink.) The two quantities involved in contribution analysis of birth rate, fecundity and proportion of adults, are ratios, the former being the ratio of the number of eggs to the number of adults and the latter, of the number of adults to the number of all animals (juveniles plus adults). A ratio does not change with proportional changes in the numerator and the denominator. Consequently, both fecundity and proportion of adults are unlikely to be affected by temperature, and thus, temperature is hardly a confounding factor in interpreting the results of contribution analysis.

We have found that the ratio of contributions in the lake closely matches the experimental value in 1 year of observation and differs from it in a predictable way in the other. Our next finding is that, in both years, *R*‐values indicative of strong bottom‐up effects are obtained at the minimum time interval of 6–12 days, which includes two to four sampling intervals over which *R* needs to be averaged. Thus, contribution analysis of birth rate, and the ratio of contributions based on it, has successfully passed the field test and, most probably, can be used to identify strong bottom‐up effects in natural zooplankton populations. Furthermore, the temporal resolution of the method is found to be as short as 2–4 sampling intervals, or 6–12 days.

The temporal resolution of contribution analysis of birth rate revealed in this study has a clear biological meaning. In Table 2, we compiled literature data on the time to maturity (from hatching to maturity) for a number of *Bosmina* species or populations. The time to maturity *D*_m_ adjusted to the average lake temperature in 2013 is found to be 3.7–9.2 days (Table 2, all rows except the last one; temperature for 2013, and not for 2012, is taken because detailed body‐size measurements, to be used below, are available only for that year). We also calculated *D*_m_ directly for our *Bosmina* population using regression equations that relate *D*_m_ to water temperature and body size in cladocerans (Gillooly, 2000; Gillooly et al., 2002); this gives 5.3–5.7 days (Table 2, the last row), which lie within the range of the literature data. We feel more confidence in the latter values and rely on them below, because they are more relevant to our population—not only in terms of temperature but also in terms of body size. Summing the time to maturity of 5.3–5.7 days and the egg development time, which is 3.5 days (calculated from the average temperature of 17.6°C in 2013 using an equation in Bottrell et al., 1976, see Section 2), gives the generation time of our *Bosmina* population to be 8.8–9.2 days. It turns out that the temporal resolution of the method, 6–12 days, is close to the generation time of the species, about 9 days.

**TABLE 2:** Time to maturity of *Bosmina* and additional relevant information.

Species	Experimental data (except for our data in the last row)				Calculated
	Body length at maturity, mm	Clutch size, eggs per clutch	Water temperature, °С	*D*_*m*1_, days	*D*_*m*2_, days (at 17.6°С)
*Bosmina coregoni* ^a^	0.38–0.40	8	15	8–10	6.4–8.1
*Bosmina coregonia* ^a^	0.32	8	20	3	3.7
*Bosmina longirostris* ^b^	0.38	1.7	11	14–16	8.0–9.2
*Bosmina longirostris* ^b^	0.34	1.1	21	3–4	4.0–5.3
*Bosmina coregoni* ^c^	0.42 ± 0.02 (±SD)	NA	15	7	5.6
*Bosmina longirostris* ^c^	0.38 ± 0.02 (±SD)	NA	15	7	5.6
*Bosmina longispina maritima* ^d^	0.39 ± 0.02 (±SD)	1.3 ± 0.5 (±SD)	10	10.6 ± 1.0 (±SD)	5.0–6.1
*Bosmina longispina maritima* ^d^	NA	2.8 ± 0.8 (±SD)	15	5.9 ± 0.2 (±SD)	4.6–4.9
*Bosmina longirostris* ^e^	0.27 ± 0.01 (±SE)	1.1 ± 0.1 (±SE)	20	5.8 ± 0.8 (±SE)	6.1–8.0
Our data, 2013	0.25 ± 0.02 (±SD)	1.0 ± 0.2 (±SD)	17.6	NA	5.3–5.7

*Note*: *D*_*m*1_, time to maturity measured in the experiments (literature data); *D*_*m*2_, time to maturity adjusted to 17.6°C, which is the average lake temperature in 2013. For the experimental data, *D*_*m*2_ is calculated on the basis of *D*_*m*1_ using a temperature correction from the Arrhenius equation: *D*_*m*2_ = *D*_*m*1_ exp(6962.98(1/(17.6 + 273) – 1/(*T*_*c*1_ + 273))) where *T*_*c*1_ is temperature in the experiments in degrees Celsius, and 6962.98 is the ratio of the activation energy (0.6 eV) to Boltzmann's constant (8.617 × 10^−5^ eV K^−1^) expressed in kelvin (K) (based on Gillooly et al., 2002; all rows except the last one); range of *D*_*m*2_ is calculated from the range of *D*_*m*1_ which is shown either directly or as mean ± SD or mean ± SE, depending on what (if any) is provided in the original publications. For our *Bosmina* population, *D*_*m*2_ is calculated from the water temperature and body mass, either on the basis of the equation given in Gillooly et al. (2002: figure 4; first value in the last row of *D*_*m*2_) or on the basis of the equation for postembryonic development time (Gillooly, 2000; second value in the last row of *D*_*m*2_). Body mass is calculated as *M* = 26.6 *L* ^3.13^ where *M* is dry body mass in μg, *L* is body length in mm (Dumont et al., 1975). Body length and clutch size presented in the table serve to generally characterize animals and trophic conditions in the experiments and in our lake. Our data refer to 2013 only because in 2012 *Bosmina* were not accurately measured for body length.

Abbreviation: NA, data not available.

^a^ Semenova (1968).

^b^ Bhajan and Hynes (1972).

^c^ Vijverberg (1980).

^d^ Kankaala and Wulff (1981).

^e^ Urabe (1991) (data at food concentration of 0.05 mg C L^−1^, which most closely corresponds to the characteristics of *Bosmina* in the present study).

The above result has an interesting and important consequence: The temporal resolution of the method seems comparable to the lifetime of a single strong interaction. There are two arguments and a guess to support this assertion. The first argument is based on the simple observation made by planktonologists that in temperate lakes in the course of the season zooplankton populations often go through two or three population maxima and minima, so each rise or fall would last one or two or perhaps 3 weeks. The second argument, based on modeling (Higgins et al., 1997; Ricker, 1954), is that the period of population oscillations tends to be twice the generation time; hence, one generation time approximates the duration of a population increase or decline. Given that the generation time in cladocerans, a major component of zooplankton, is about one or two weeks (see, e.g., Table 2 for the time to maturity in *Bosmina*, but note that it is shorter than the generation time), and may be even longer in the case of food shortages (Romanovsky, 1984), the second argument leads to approximately the same duration of monotonic population changes as does the first. Our guess is that, often, there may be only one major driving force, for example, food or predators, responsible for a single population growth or decline event (cf. driver‐response relationships in time series, Ryo et al., 2019). This reasoning suggests that contribution analysis of birth rate has sufficient temporal resolution to detect the effect of food on major population‐dynamics events just “on the fly,” right at the time that the effect occurs.

Contrary to the expectation that the length of monotonic segments of population change is about the generation time, our *Bosmina* populations continuously grew several times longer (Figure 1). Apparently, from late June to early August in both years of study, the lake's system was in the state of a prolonged biological spring, which is evidenced by the similarity between the population growth curves in this northern lake and those observed during the calendar spring in temperate lakes as described by the PEG model (Sommer et al., 1986, 2012). Given that contribution analysis of birth rate allows one to detect the main factor of population dynamics on relatively short intervals of about the generation time, it will be all the more able to do so on longer intervals. This simply follows from the fact that on intervals longer than the generation time the *R* curves generally plateau—and just at the expected values (see Figure 4).

Our *Bosmina* populations experienced only one strong effect, that of food, and did not exhibit major population fluctuations. Usually, and especially in more complex systems than the one studied here, the population of interest will experience both bottom‐up and top‐down effects and show pronounced population fluctuations. How then would we apply the method? A possible procedure could be as follows: consider a period of monotonic population growth (rise or decline), collect samples often enough to have, say, five samples during that period (four sampling intervals), calculate *R*, for example, for all consecutive pairs of sampling intervals, that is, for the first and the second interval, the second and the third, and the third and the fourth (alternatively, for all consecutive triples, which gives only two values of *R*), and then calculate the average over those values of *R*. This procedure would be exactly the same as used in the present study and might therefore provide instructive *R*‐values. The assumption it relies on, which repeats our guess stated above, is that a single population growth or decline event is associated with only one major trophic force, either bottom‐up or top‐down.

The results of this study are obtained with the use of the Edmondson–Paloheimo model (Edmondson, 1968; Paloheimo, 1974). It has been repeatedly shown that the model relies on rather general assumptions and approximates quite well birth rate in zooplankton, especially in cladocerans (e.g., Hairston, 2015; Mooij et al., 2003; Polishchuk, 1986). There are a number of mathematical derivations of the model (the most elegant and general one is due to Voronov, 1991), which all link birth rate to time intervals equal to *D*, the egg development time, thus implying that an interval of length *D* is an adequate sampling interval to estimate birth rate (Polishchuk, 1982, 1986). Given that the egg development time at normal summer temperatures is about 3 or 4 days, this is the reason why in this study sampling intervals are chosen to be just that length. The possibility of identifying strong bottom‐up effects on relatively short time intervals demonstrated in this study comes of course from the power of a general approach, Caswell's contribution analysis. We emphasize however that to no lesser extent does it come from the power of a particular model to which contribution analysis has been applied, the Edmondson–Paloheimo model, especially when it is used at sampling intervals of about the egg development time, as is the case here.

There are two general methods commonly used to assess the strength of ecological interactions: One is based on matrices of partial derivatives (Jacobian matrices; de Ruiter et al., 1995; Novak et al., 2016) and the other makes use of multivariate autoregressive (MAR) models (Gabaldón et al., 2019; Hampton et al., 2006; Huber & Gaedke, 2006; Ives, 1995; Ives et al., 1999). Contribution analysis of birth rate involves partial derivatives and thus belongs to the former type. Table 3 provides a detailed comparison between our approach and one form of the Jacobian matrices, a community matrix, which is a matrix of partial derivatives of the population growth rates of each species with respect to population size of every species in the community (de Ruiter et al., 1995; Novak et al., 2016). The major difference is that our approach takes into account not only partial derivatives but also actual changes in the quantities of interest between sampling occasions, namely Δ(fecundity) and Δ(proportion of adults), which makes it more dynamically oriented. Thinking in terms of dynamics when “everything changes and nothing stands still” (Heraclitus) readily raises the question of the method's temporal resolution (does the method allow us to capture those changes when they occur?), which is addressed in this study. This same question can be asked for other methods but, to the best of our knowledge, it is often overlooked. Furthermore, we suggest that the temporal resolution of about the lifetime of a single strong interaction, which we came across in relation to one particular method, contribution analysis of birth rate, in a particular context, bottom‐up and top‐down effects in zooplankton, would be a generally desirable property for any method intended to identify and assess any type of strong ecological interactions, for it enables one to keep up with interaction changes. We hope that our study will not only draw attention to the problem of temporal resolution but will also stimulate the search for methods possessing the above property.

**TABLE 3:** Comparative table to show similarities and differences between the methods of assessment of the strength of top‐down and bottom‐up effects using community matrices (left section) and using contribution analysis of birth rate (right section).

Step	Interaction strength in a community matrix		Interaction strength by means of contribution analysis of birth rate	
1	A graphical representation of the effect of food (bottom‐up) and predators (top‐down) on			
	the population growth rate (*Ẋ*_1_) of a target species		the per capita birth rate (*b*) of a target species	
	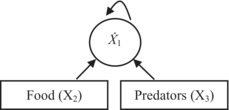		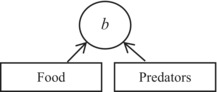	
1a	NA		A graphical representation of the effect of food and size‐selective predators on birth rate, mediated by fecundity (*F*) and proportion of adults (*A*), respectively	
			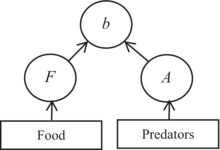	
2	A general model for			
	population growth rate		per capita birth rate	
	*Ẋ*_1_ = *f*(*X*_1_, *X*_2_, *X*_3_)		*b* = *g*(*F*, *A*, *V*)	
3	Decomposition of a change in *Ẋ*_1_ or *b* into components, each due to a change in the variable involved			
	${\dot{X}}_{1}=\frac{\partial f}{\partial X_{1}}\Delta X_{1}+\frac{\partial f}{\partial X_{2}}\Delta X_{2}+\frac{\partial f}{\partial X_{3}}\Delta X_{3}$		$\Delta b=\frac{\partial g}{\partial F}\Delta F+\frac{\partial g}{\partial A}\Delta A+\frac{\partial g}{\partial V}\Delta V$	
4	A working model for			
	population growth rate		per capita birth rate in zooplankton	
	${\dot{X}}_{1}=X_{1}\left(r_{1}+\sum_{i}a_{1i}X_{i}\right)\;i=1,2,3$		*b* = *V* ln(1 + *FA*)	
5	Interaction strength measured in terms of			
	partial derivatives		partial derivative ∙ Δ(variable) = contribution	
	Equation	Type of effect	Equation	Type of effect
	$S_{11}=\frac{\partial f}{\partial X_{1}}=r_{1}+2a_{11}X_{1}+\sum_{i}a_{1i}X_{i}\;i=2,3$	Self‐effect (not of interest here)	$\mathrm{Con}F=\frac{\partial g}{\partial F}\Delta F=\frac{\mathrm{VA}}{1+\mathrm{FA}}\Delta F$	Bottom‐up
	$S_{12}=\frac{\partial f}{\partial X_{2}}=a_{12}X_{1}$	Bottom‐up	$\mathrm{Con}A=\frac{\partial g}{\partial A}\Delta A=\frac{\mathrm{VF}}{1+\mathrm{FA}}\Delta A$	Top‐down
	$S_{13}=\frac{\partial f}{\partial X_{3}}=a_{13}X_{1}$	Top‐down	$\mathrm{Con}V=\frac{\partial g}{\partial V}\Delta V=\Delta V\cdot \text{In}\left(1+\mathrm{FA}\right)$	Temperature effect (not of interest here)

*Note*: The methods are divided into five (community matrices) or six (contribution analysis) steps, as indicated. The main similarity between the methods is that both employ partial derivatives to measure interaction strengths, and the main difference is that contribution analysis, after Caswell (1989), additionally accounts for actual changes in the variables considered so that the strength of the effect is expressed as the partial derivative with respect to a given variable times the actual shift in that variable (Step 5). An additional difference is that contribution analysis of birth rate involves population characteristics (fecundity and proportion of adults) that are intermediate between the environmental factors (food and predation, respectively) and the population's response (Step 1a; this step is not available, NA, in the community matrices method). *Community matrices section*. *X*_1_, *X*_2_, and *X*_3_ are the abundances of a target species, food, and predators, respectively. Arrows indicate the effect, including a self‐effect (Step 1). In the left‐hand side of the expression for decomposition (Step 3), there is *Ẋ*_1_ rather than Δ*Ẋ*_1_ because the expansion is performed around the equilibrium point at which *Ẋ*_1_ = 0. The model shown at Step 4 is a generalized Lotka–Volterra model (Yodzis, 1989) commonly used in the context of community matrices (Novak et al., 2016). *Contribution analysis of birth rate section*. Arrows indicate the effect (Steps 1 and 1a). Predators are assumed to be size‐selective, as is often the case in zooplankton, hence the effect of predators on the proportion of adults *A* and through *A* on per capita birth rate *b* (Step 1a). The model shown at Step 4 and used in this study and elsewhere (Polishchuk, 1995; Polishchuk et al., 2013) is the Edmondson–Paloheimo model of birth rate in zooplankton (Edmondson, 1968; Paloheimo, 1974). Along with fecundity and proportion of adults, the model includes the egg development rate *V*, which is the reciprocal of the egg development time. The latter is known to largely depend on temperature; its effect on birth rate, and hence the effect of temperature, is not considered here. More information is given in the body of the table.

It is a popular belief that Nature is extremely complex, or, as some people would put it, “everything depends on everything else.” This discouraging picture may be misleading, though. One of the reasons for this misconception is that the methods used often do not have sufficient temporal resolution or the problem of resolution is simply ignored. The lack of resolution will automatically lead to averaging over long time intervals, resulting in multiple factors contributing roughly equally to the process of interest and thus indeed giving the impression, a false one, that everything depends on everything else. The situation may be different on short time intervals, where just a single strong interaction is likely to occur. But how short do those short intervals need to be? The answer “the shorter the better” seems neither practical nor heuristically useful. Let's put it differently: What is a time interval, not too short but a reasonable one, sufficient to identify strong interactions? The answer proposed in this paper is that, conceptually, its length equals the lifetime of a single strong interaction. Furthermore, in the context of population dynamics, the interval can be defined more precisely: It is about the generation time of the species of interest. This is because the periods of population increase or decline are often comparable to the species' generation time, and each of those increase or decline events may be associated with a single major driving force or, equivalently, a single strong interaction. We came up with these ideas inductively while exploring one particular method, contribution analysis of birth rate in zooplankton, but believe they are of general interest. The method's temporal resolution is found to be approximately the generation time of the species to which it has been applied. Importantly, the method does not allow us to get rid of averaging altogether; some averaging is necessary, since an interval of the length of generation time must accommodate two to four sampling intervals over which the averaging is performed. Given our species' generation time, this means that each sampling interval is to be 3–4 days, which is the case in this study. It remains to be seen whether, and to what extent, averaging is a general requirement, that is, how the necessity for temporal resolution interacts with the need for temporal averaging. To conclude, our work shows that contribution analysis of birth rate is a promising tool to identify strong trophic interactions in zooplankton. In a broader perspective, this study suggests that dynamically oriented methods with a temporal resolution of about the lifetime of a single strong interaction may be key in disentangling strong interactions in ecological communities and thus in solving one of the main issues in ecology.

## AUTHOR CONTRIBUTIONS

**Leonard V. Polishchuk:** Conceptualization (lead); data curation (supporting); funding acquisition (lead); methodology (lead); software (lead); validation (equal); visualization (equal); writing – original draft (equal); writing – review and editing (lead). **Anna A. Kasparson:** Conceptualization (supporting); data curation (lead); funding acquisition (supporting); methodology (supporting); software (supporting); validation (equal); visualization (equal); writing – original draft (equal); writing – review and editing (supporting).

## FUNDING INFORMATION

The work was carried out within the framework of the Interdisciplinary Scientific and Educational School “The Future of the Planet and Global Environmental Changes,” Lomonosov Moscow State University, and the MSU theme no. 121032300124‐1, with partial financial support of the Russian Foundation for Basic Research, project no. 18‐04‐01143.

## CONFLICT OF INTEREST STATEMENT

No conflicts of interest.

## Supporting information


Appendix S1

## Data Availability

The data that support the findings of this study are openly available in Figshare at https://doi.org/10.6084/m9.figshare.21268512. A QBasic computer code to calculate contributions, file “ConBasic.BAS,” is available in Appendix S1. Two more supplementary files are “Datain.txt,” which is an example of an input data file, and “CONTS.txt,” which contains the output data, that is, contributions, calculated from the input data. The input file shows how input data must be arranged, and the output file can be used to check whether the computer code works correctly. For further analysis of the contribution results, the CONTS.txt file can be converted into an Excel spreadsheet. The fourth supplementary file, “Description_of_files_used_to_calculate_contributions,” as the name suggests, details the content of the above three files.
