# Supplementary material for: Temporal resolution of birth rate analysis in zooplankton and its implications for identifying strong interactions in ecology
Source: Ecol Evol. 2023 Jul 24;13(7):e10341. doi: 10.1002/ece3.10341 (PMC10366112; doi:10.1002/ece3.10341)
Supplement: Supplementary file 1 — Appendix S1 [file ECE3-13-e10341-s001.zip › Description_of_files_used_to_calculate_contributions.docx]

**Description of files used to calculate contributions**

File **ConBasic.bas** contains a QBasic code used to calculate, by numerical integration, the contributions of changes in egg development rate *V*, fecundity *F* and proportion of adults *A* to the resulting change in birth rate. The contributions are calculated on a per sampling interval basis.

File **Datain.txt** is an example of an input file to show how the input data must be arranged. It contains the number of data points (samples) and four datasets: *V*, *A*, *F* and *T*, in that order, where *T* is the interval between successive samples; the datasets are arranged in column form and placed one below another. The number of rows in each of the *V*, *A* and *F* datasets equals the number of samples; the number of rows in the *T* set is one fewer.

File **CONTS.txt** is an output file for the **Datain.txt** input file. This file is provided to check whether **ConBasic.bas** works correctly. The description of the content of **CONTS.txt** is given in the body of the **ConBasic.bas** script. For further analysis of the results, the **CONTS.txt** file can be converted into an Excel spreadsheet.
